# Low-dose trimethoprim-sulfamethoxazole treatment for Pneumocystis pneumonia: a systematic review and meta-analysis

**DOI:** 10.3389/fphar.2024.1422490

**Published:** 2024-11-13

**Authors:** Hui-Bin Huang, Yi-Bing Zhu, Da-Xing Yu

**Affiliations:** Department of Critical Care Medicine, Guang’anmen Hospital, China Academy of Chinese Medical Sciences, Beijing, China

**Keywords:** Pneumocystis jirovecii pneumonia, trimethoprim-sulfamethoxazole, adverse event, mortality, meta-analysis

## Abstract

**Background:**

The recommended standard treatment for Pneumocystis jirovecii pneumonia (PJP) is high-dose trimethoprim-sulfamethoxazole (TMP-SMX) (15–20 mg/kg/d TMP). However, the standard regimen may cause a high incidence of dose-related adverse events (AEs). Therefore, we aimed to conduct a systematic review and meta-analysis to evaluate the efficacy and safety of low-dose TMP-SMX regimens (<15 mg/kg/d of TMP) compared with the standard regimen in patients with PJP.

**Methods:**

We searched PubMed, Embase, and the Cochrane database for relevant articles from inception to 10 March 2024. Studies were included if they focused on PJP patients receiving a low-dose TMP-SMX regimen compared with a standard regimen. The primary outcome was mortality. We assessed study quality and performed subgroup analysis and sensitivity analysis to explore potential heterogeneity among the included studies.

**Results:**

Seven studies were included. Overall, the low-dose regimen significantly reduced the risk of mortality (odds ratio [OR] = 0.49; 95% CI, 0.30–0.80; *I* ^2^ = 16%; *P* = 004). This finding was confirmed in further sensitivity and subgroup analyses. The low-dose regimen also significantly reduced total AEs (OR = 0.43; 95% CI, 0.29–0.62; *I* ^2^ = 0%; *P *< 0.0001), and improved the incidence of most specific AEs (ORs ranged from 0.13 to 0.89). In addition, the low-dose regimen had significantly more patients completing the initial regimen (*P* = 0.002), fewer patients requiring dose reductions (*P* = 0.04), and almost significantly fewer patients requiring a switch to a second-line regimen (*P* = 0.06).

**Conclusion:**

The limited available evidence suggests that a low-dose TMP-SMX regimen significantly reduced mortality and total AEs in PJP patients. Thus, it is one of the potentially promising therapies to PJP and more high-quality and multi-center randomized trials should be conducted in the future.

## Introduction

Pneumocystis jiroveci pneumonia (PJP) is a serious respiratory disease common in immunocompromised patient populations. The overall mortality rate for PJP is 6%–11% (Mansharamani et al., 2000), depending on the underlying disease, comorbidities, and degree of immunosuppression. Notably, non-HIV PJP patients had a higher mortality rate (30%–60%) than HIV PJP patients (10%–20%) (Thomas, 2004; Ward and Donald, 1999). In addition, non-HIV PJP has growing in recent years with the increasing number of patients receiving transplantation, immunosuppressants, antitumor chemotherapy and prolonged corticosteroids.

Trimethoprim-sulfamethoxazole (TMP-SMX) can treat PJP by interfering with folate metabolism (Lane et al., 1997). For many years, TMP-SMX has been considered the first-line treatment for PJP, with guidelines recommending a standard dosage of TMP 15–20 mg/kg/d combined with SMX 75–100 mg/kg/d by (Fishman et al., 2019; Mofenson et al., 2009). However, the standard dosing regimen can cause serious adverse events (AEs) and drug toxicity in over 30% of PJP patients regardless of response rates on TMP-SMX, including rash, drug fever, neutropenia, renal insufficiency, electrolyte disturbances, and hepatotoxicity (Medina et al., 1990). Once these AEs occur, it is difficult for patients to continue treatment; and clinicians often have to reduce the dose, discontinue the treatment regimen, or switch to other therapies.

In recent years, a low-dose TMP-SMX regimen (<15 mg/kg/d or lower TMP) has been used for the PJP treatment and has shown promising results. Several studies reported that the low-dose regimen had comparable efficacy and fewer AEs compared with the standard regimen (Creemers-Schild et al., 2016; Kosaka et al., 2017; Nakashima et al., 2018; Ohmura et al., 2019; Ji et al., 2021). In these studies, fewer patients who received a low-dose regimen discontinued treatment or switched to an alternative therapy. However, the sample sizes of these studies were small, and the findings were inconsistent. This may be related to the differences in patient populations, dosing regimens, treatment strategies, and study designs among the studies (Creemers-Schild et al., 2016; Kosaka et al., 2017; Nakashima et al., 2018; Ohmura et al., 2019). Therefore, the efficacy and safety of the low-dose TMP-SMX strategy for patients with PJP still lack sufficient evidence to support its clinical implementation.

Recently, several studies have been published on the use of low-dose TMP-SMX regimens in PJP patients (Gu et al., 2022; Hammarström et al., 2023; Nagai et al., 2024). Therefore, with the power of meta-analysis, we aimed to conduct a systematic review and meta-analysis to explore the efficacy and safety of low-dose TMP-SMX strategy in this patient population. We also performed subgroup analyses and sensitivity analyses to examine potential confounders.

## Methods

The current study followed the guidelines of the PRISMA statement (Page et al., 2021) (Supplementary File S1) and the protocol has been registered on the International Platform of Registered Systematic Review and Meta-analysis Protocols database (Registration number: INPLASY202440085).

### Data sources and search strategy

Two reviewers (H-BH and Y-BZ) independently searched the following scientific databases: PubMed, EMBASE, and Cochrane databases from inception to 10 March 2024. The search strategy included MeSH terms and keywords for “dose,” “sulfamethoxazole,” “SMX-TMP,” “trimethoprim-sulfamethoxazole,” “Pneumocystis jirovecii pneumonia,” and “Pneumocystis carinii pneumonia” without language restriction. Details of the search strategy are summarized in Supplementary File S2. Moreover, we screened the references of included studies and retrieved reviews to avoid omitting any relevant studies.

### Inclusion and exclusion criteria

Studies were included if they met the following inclusion criteria: (1) study design: randomized clinical trials (RCT) or observational study with two-arm comparisons; (2) adult patients (>18 years old) with PJP; (3) patients received different doses of TMP-SMX (i.e., low-, standard, or high-dose) as defined by authors; (4) studies should report any efficacy or safety outcomes. The following studies were excluded: studies enrolling neonatal, children, and pregnant women; studies focusing on reduced-dose TMP-SMX in single-arm; studies published only in the abstract, meeting reports, commentaries, reviews, or protocols; and studies with specific data unavailable.

### Data extraction and outcomes

Relevant data were extracted from eligible articles, including the study characteristics (author and year, study design, sample size, and country), patient characteristics (age, gender, patient population), dosing regimens, adjunctive therapies (i.e., corticosteroid or combinations of any other anti-PJP drugs), and predefined outcomes. The primary outcome was the all-cause mortality at the longest follow-up available during the study period. Secondary outcomes were adverse events (defined by each author) and implementation of the initial TMP-SMX regimen (i.e., completion of initial treatment, switch to second-line regimen, and dose reduction). Disagreements were identified and resolved by consensus.

### Quality assessment

The quality of each included study was assessed by two of the reviewers independently using the Newcastle-Ottawa scale (NOS) (Stang, 2010). We evaluated publication bias by visual inspection funnel plots when at least ten studies were included in this meta-analysis. Disagreements were resolved by detailed discussion or consulting a third author (D-XY).

### Statistical analysis

The results from all relevant studies were combined to estimate the pooled odds ratio (ORs) and associated 95% confidence intervals (CIs) for dichotomous outcomes. For the continuous outcomes, we estimated mean differences (MD) and 95% CIs as effective results. We conducted meta-analyses by pooling relevant studies to analyze each predefined outcome. In the current meta-analysis, we defined a low dose of TMP <15 mg/kg/d. To explore the potential influences of the primary outcome, we performed sensitivity analyses on primary outcome (mortality) by pooling studies only focusing on: (a) adjunctive steroids use; (b) non-HIV infection; (c) sample size >50; (d) TMP-SMX as the only initial drug; (e) low-dose of TMP < 10 mg/kg/d; and (f) low-dose of TMP < 15 mg/kg/d. Additionally, subgroup analyses were conducted separately by pooling studies based on (1) statistical analysis: fixed-effects mode or random-effects mode; (2) follow-up: short-term (30-day, ICU stay, or hospital stay) or long-term (≥90-day) mortality; (3) Diagnosis criteria: included 1,3-β-D-glucan test or non-included 1,3-β-D-glucan; and (4) mortality prevalence: ≥20%, or <20% (calculated according to the standard dose group).

To test for heterogeneity, we employed the *I* ^2^ statistic. We consider the value of *I* ^2^ < 50% and *I* ^2^ > 50% to indicate low and high heterogeneity, respectively. For our analysis, we utilized the Mantel-Haenszel method (Higgins et al., 2003), applying a fixed-effect model when *I* ^2^ > 50% and a random-effect model when *I* ^2^ < 50%. We set the significance level for *P* values at 0.05. All analyses were executed using Review Manager (version 5.4).

## Results

### Searching results

**FIGURE 1:**
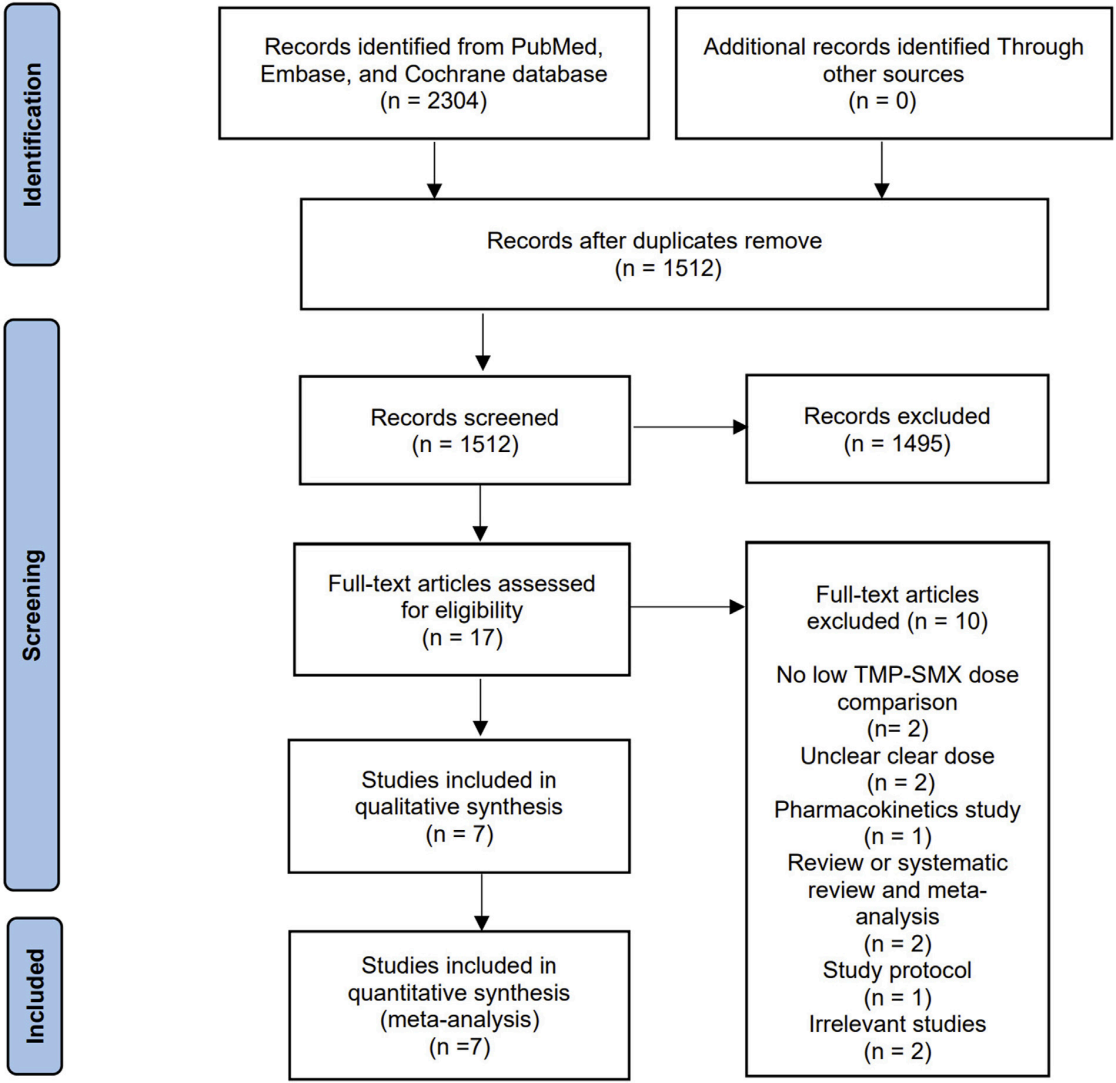
Flow chart of literature selection.

Our electronic search yielded 2,304 records from the predefined databases. We performed a deduplication process and obtained 1,512 records, out of which 1,495 were excluded based on title and abstract screening. After conducting a thorough full-text review of the remaining 17 studies, we excluded 10 articles, as summarized in Additional File S3. Consequently, we included seven retrospective cohort studies in the final analysis (Creemers-Schild et al., 2016; Kosaka et al., 2017; Nakashima et al., 2018; Ohmura et al., 2019; Gu et al., 2022; Hammarström et al., 2023; Nagai et al., 2024; Chang et al., 2016) (Figure 1).

### Study characteristics and quality assessment

Table 1 describes the main characteristics of the included studies. The studies were conducted between 2016 and 2024 and involved 532 patients (Kosaka et al., 2017; Nakashima et al., 2018; Ohmura et al., 2019; Gu et al., 2022; Hammarström et al., 2023; Nagai et al., 2024; Chang et al., 2016). Four included studies were multicenter studies (Kosaka et al., 2017; Ohmura et al., 2019; Hammarström et al., 2023; Nagai et al., 2024). One study specifically examined HIV-infected patients (Chang et al., 2016) while the others focused on individuals with various medical conditions, such as immunological and rheumatic diseases, solid or hematologic malignancies, interstitial lung diseases, and more. These studies were conducted in different countries, including Japan (Kosaka et al., 2017; Nakashima et al., 2018; Ohmura et al., 2019; Nagai et al., 2024), China (Gu et al., 2022; Chang et al., 2016), and Sweden (Hammarström et al., 2023). The definitions and outcomes of mortality and definitions of low-dose TMP of each study among the included studies were summarized in the Additional File S4. The severity of respiratory failure the patients differed among the included studies, which were summarized in Additional File S5.

**TABLE 1:** Characteristics of the included studies in the current systematic review and meta-analysis.

Study	Country	Study design	Underlying condition	Sample, LD/CTRL	Dosing regimens, mg/kg/day, TMP	Steroid used, % LD/CTRL	Age, year, LD/CTRL	Male, %, LD/CTRL	Predefined outcomes	Follow-up
Chang et al. (2016)	China	R, SC	HIV: ⑧	25/27	<15; ≥15	100/100	34.6^a^	96.2^a^	AE	30 days
Gu et al. (2022)	China	R, SC	Non-HIV: ⑥	10/10	8; 15	NA	40/48.5	10/80	Mortality, AE	Hospital
Hammarström et al. (2023)	Sweden	R, MC	Non-HIV: ①	80/33	7.5–15; 15–20	72.5/81.8	68/67	76/58	Mortality, AE	8, 30 days
Kosaka et al. (2017)	Japan	R, MC	Non-HIV: ①②④⑤⑦	41/36	<15; 15–20	85.7/80.6	65/67	68.3/52.8	Mortality, AE	90 days
Nagai et al. (2024)	Japan	R, MC	Non-HIV: ①⑦	55/81	<12.5; 12.5–20	89.1/86.4	71/71	51/41	Mortality, AE	30, 180 days
Nakashima et al. (2018)	Japan	R, SC	Non-HIV: ①②③④⑤⑦	24/29	4–10; 10–20	95.8/89.7	72/73	54.2/72.4	Mortality, AE	30, 180 days
Ohmura et al. (2019)	Japan	R, MC	Non-HIV: ②	22/30/29	≤10; 10–15; 15–20	77.3/63.3/72.4	67/64/66	31.8/30/17.2	Mortality, AE	30, 180 days

^a^ The total cohort.

① hematologic malignancies; ② connective tissue disease; ③ immunosuppressive drugs for non-malignant disease; ④ idiopathic interstitial pneumonia; ⑤ collagen vascular disease; ⑥ renal transplantation; ⑦ solid tumors; ⑧ Acquired Immune Deficiency Syndrome (AIDS).

AE, adverse events; CTRL, control group; HIV, human immunodeficiency virus-infected; LD, low-dose group; MC, multicentre; NOS, Newcastle-Ottawa scale; R, retrospective; SC, single-center; TMP, trimethoprim.

We evaluated the risk of bias in each included study using the NOS method (Additional File S6). The quality of the observational studies was moderate to high. Assessment of publication bias using visually inspecting funnel plots showed no potential publication bias in the included studies (Additional File S7).

### Primary outcome

**FIGURE 2:**
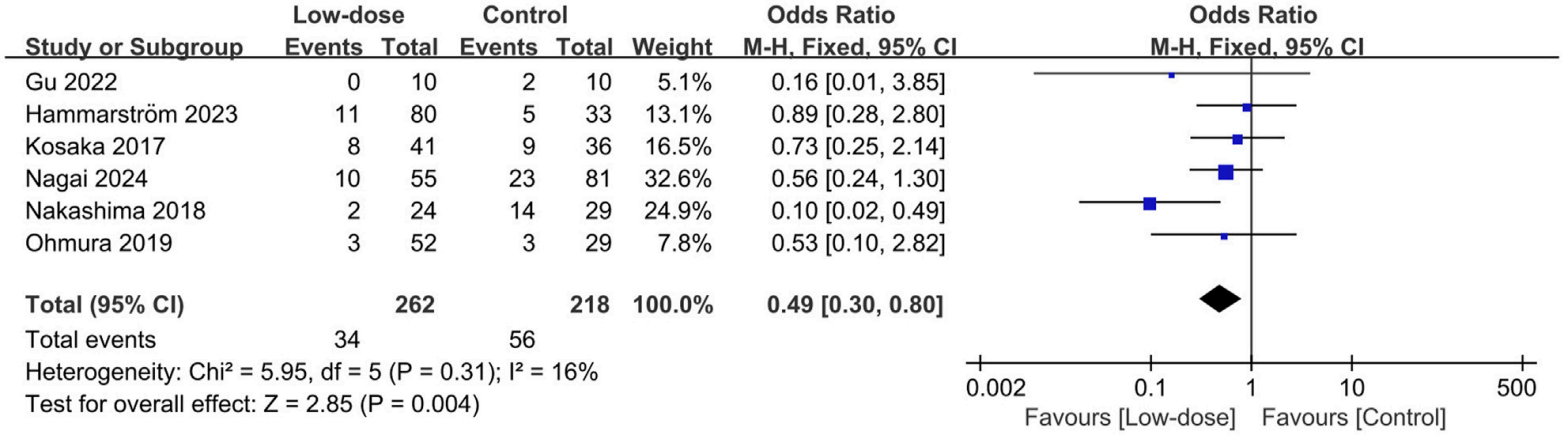
Forest plots of the effect of low-dose TMP-SMX regimen on mortality rate in treating patients with PJP.

All causes of mortality was reported in six studies (Creemers-Schild et al., 2016; Kosaka et al., 2017; Nakashima et al., 2018; Ohmura et al., 2019; Gu et al., 2022; Hammarström et al., 2023; Nagai et al., 2024). Of these, 262 patients received low-dose SMX-TMP, and 34 died (12.9%), compared with 218 patients in the control group, of whom 56 died (25.9%). We found that the low-dose TMP-SMX regimen was associated with a significantly reduced mortality rate compared with the standard regimen (OR = 0.49; 95% CI, 0.30–0.80; *I* ^2^ = 16%, *P* = 0.004) (Figure 2). Subsequently, we performed the sensitivity analyses to investigate the sources of heterogeneity, and found that when only adjunctive steroids use or non-HIV infection or sample size >50 or SMX-TMP as the only initial drug or <10 mg/kg/day in the low-dose regimen or <15 mg/kg/day in the low-dose regimen were considered, there was a significant reduction in the low-dose regimen group (*P* values ranged from 0.008 to 0.05, *I* ^2^ from 0% to 26%). Subgroup analyses were subsequently conducted on predefined key study characteristics and clinical factors. In general, all the subgroup analyses confirmed a consistent reduction in mortality in the low-dose group, except for studies that included the 1,3-β-D-glucan or studies with a mortality prevalence less than 10% (Table 2).

**TABLE 2:** Sensitivity and subgroup analyses of the effect of low-dose SMX-TMP on mortality.

Study characteristics		Studies number	Number of patients	Event in low-dose group	Event in control group	Odds ratio (95 % CI)	I^2^	p
Sensitivity analyses								
	Adjunctive steroids	5	460	34 of 252 (12.6%)	54 of 208 (25.5%)	0.51 (0.31, 0.84)	26%	0.008
	Non-HIV infection	6	480	34 of 262 (12.6%)	56 of 218 (25.5%)	0.49 (0.30, 0.80)	16%	0.004
	Sample size >50	5	460	34 of 252 (12.6%)	54 of 208 (25.5%)	0.51 (0.31, 0.84)	26%	0.008
	SMX-TMP as the only initial drug	5	460	34 of 252 (12.6%)	54 of 208 (25.5%)	0.51 (0.31, 0.84)	26%	0.008
	<10 mg/kg/day in low-dose group	3	154	2 of 56 (12.8%)	20 of 98 (27.1%)	0.13 (0.04, 0.50)	0%	0.008
	<15 mg/kg/day in low-dose group	6	480	34 of 262 (14.5%)	56 of 218 (24.7%)	0.49 (0.26, 0.95)	16%	0.05
Subgroup analyses								
Statistical analysis	Randomized-effects modes	6	480	34 of 262 (12.6%)	56 of 218 (25.5%)	0.51 (0.29, 0.91)	16%	0.02
	Fixed-effects modes	6	480	34 of 262 (12.6%)	56 of 218 (25.5%)	0.49 (0.30, 0.80)	16%	0.004
Follow-up	Short-term	6	480	18 of 221 (8.1%)	31 of 182 (17.0%)	0.42 (0.22, 0.82)	10%	0.01
	Long-term	4	347	23 of 172 (13.4%)	49 of 175 (28%)	0.45 (0.21, 0.95)	33%	0.04
Diagnosis	Included G-test	2	217	13 of 107 (14.5%)	26 of 110 (24.7%)	0.55 (0.26, 1.17)	0%	0.12
	Not included G-test	4	263	21 of 155 (10.2%)	30 of 108 (26.9%)	0.45 (0.24, 0.85)	35%	0.01
Mortality prevalence^a^	≥20 %	4	286	20 of 130 (8.3%)	48 of 156 (48.3%)	0.42 (0.24, 0.76)	39%	0.004
	<20 %	2	101	14 of 132 (13.0%)	8 of 62 (23.0%)	0.76 (0.30, 1.93)	0%	0.56

^a^ Calculated according to the control group.

HIV, human immunodeficiency virus-infected; LD, low-dose.

### Secondary outcomes

**FIGURE 3:**
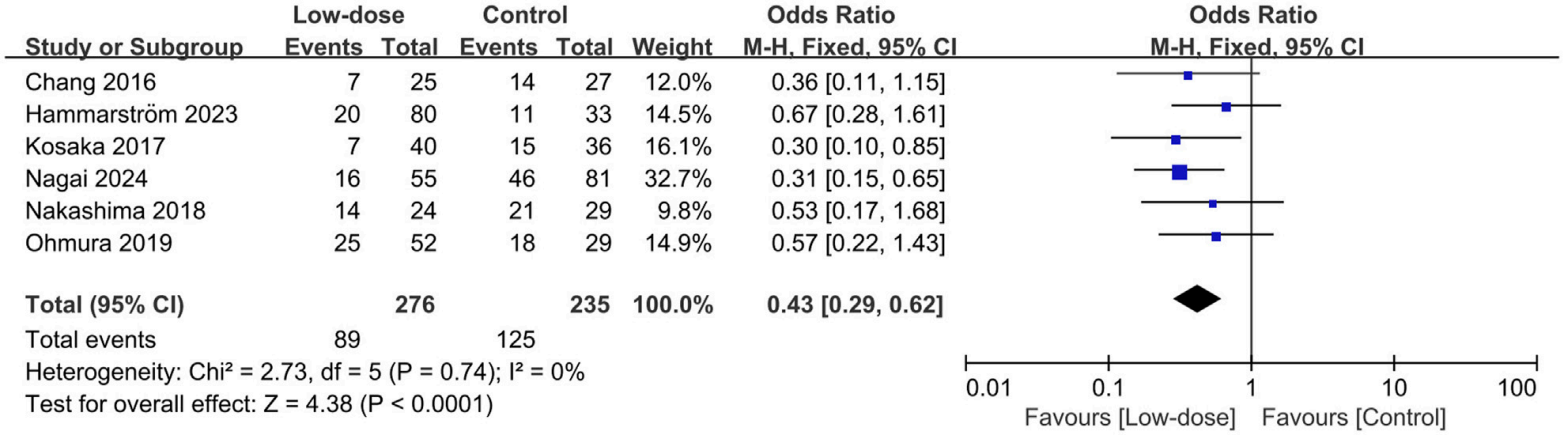
Forest plots of the effect of low-dose TMP-SMX regimen on AEs in treating patients with PJP.

**FIGURE 4:**
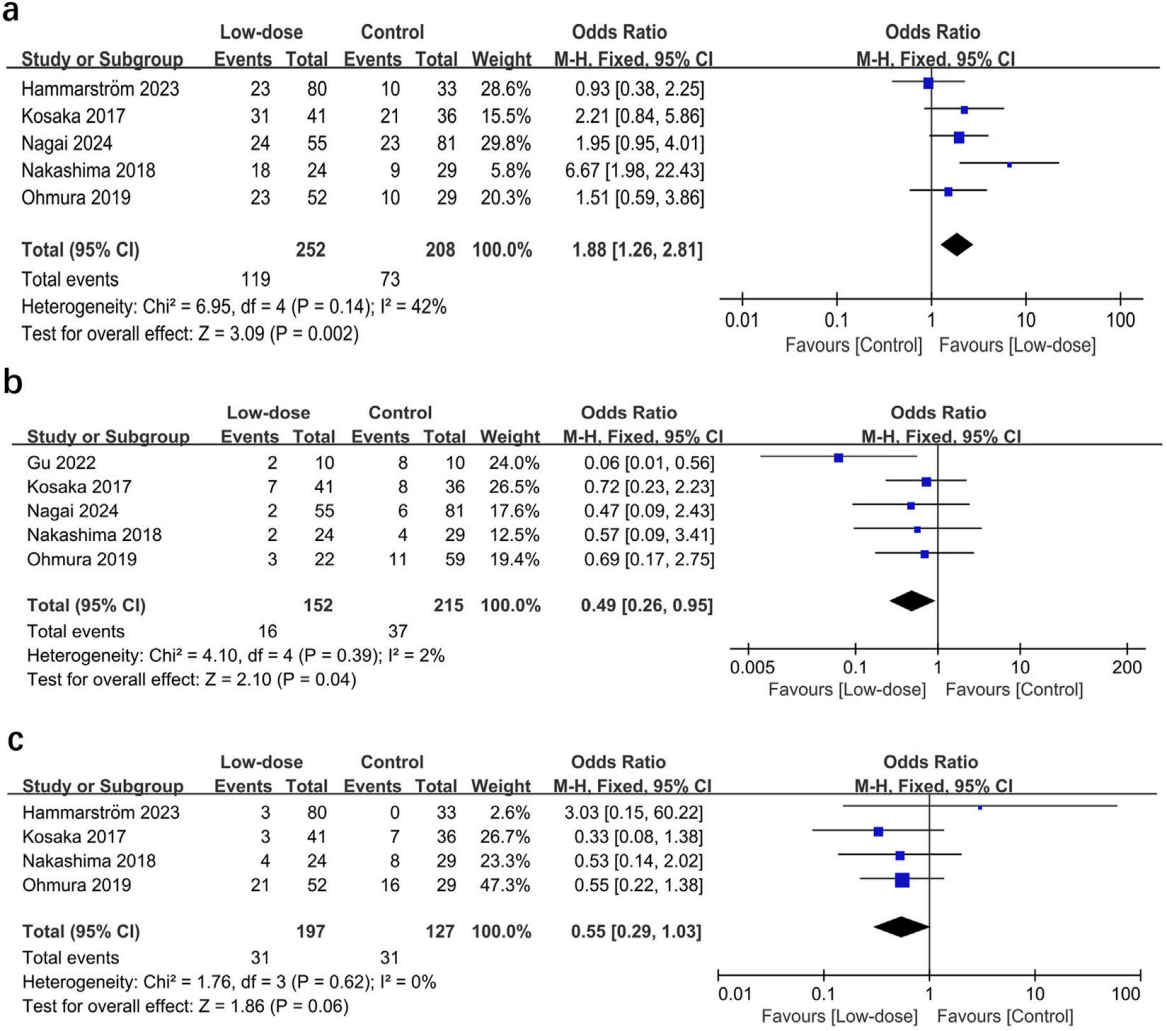
Forest plots of the effect of low-dose TMP-SMX regimen on **(A)** the incidence of patients completing initial treatment, **(B)** patients requiring dose reduction, and **(C)** patients switching to a second-line regimen.

All seven studies presented data on AEs (Kosaka et al., 2017; Nakashima et al., 2018; Ohmura et al., 2019; Gu et al., 2022; Hammarström et al., 2023; Nagai et al., 2024; Chang et al., 2016). The total incidence of AEs and the most frequently occurring AEs were summarized in the Additional File S8. The pooled estimates showed that the low-dose regimen significantly reduced the total AEs (OR = 0.43; 95% CI, 0.29–0.62; *I* ^2^ = 0%; *p* < 0.0001; Figure 3) (Creemers-Schild et al., 2016; Kosaka et al., 2017; Nakashima et al., 2018; Ohmura et al., 2019; Hammarström et al., 2023; Nagai et al., 2024; Chang et al., 2016) compared to the standard regimen. The most frequently reported AEs (reported in at least three studies) were analyzed. The low-dose regimen had a significantly reduced incidence of hyponatremia (OR = 0.35; 95% CI, 0.19–0.64; *I* ^2^ = 0%; *P* = 0.0007), anemia (OR = 0.13; 95% CI, 0.03–0.55; *I* ^2^ = 0%; *P* = 0.005), and rash (OR = 0.46; 95% CI, 0.27–0.79; *I* ^2^ = 21%; *P* = 0.005). However, the use of the low-dose regimen did not exhibit a significant beneficial effect on nausea (OR = 0.32; 95% CI, 0.04–2.72; *I* ^2^ = 50%; *P* = 0.29), leukopenia (OR = 0.38; 95% CI, 0.13–1.36; *I* ^2^ = 34%; *P* = 0.14), thrombocytopenia (OR = 0.33; 95% CI, 0.07–1.49; *I* ^2^ = 51%; *P* = 0.15), increased ALT levels (OR = 0.89; 95% CI, 0.41–1.95; *I* ^2^ = 0%; *P* = 0.77), hyperkalemia (OR = 0.53; 95% CI, 0.26–1.06; *I* ^2^ = 0%; *P* = 0.07), and renal injury (OR = 0.67; 95% CI, 0.28–1.59; *I* ^2^ = 37%; *P* = 0.37). The low-dose regimen had fewer patients requiring a switch to a second-line regimen (15.7% vs. 24.4%) and more patients to complete the initial regimen (47.2% vs. 35.1%) than the standard-dose regimen. In addition, the low-dose regimen had significantly more patients who were able to complete the initial regimen (OR = 1.88; 95% CI, 1.26–2.81; *I* ^2^ = 42%; *P =* 0.002, Figure 4A) (Kosaka et al., 2017; Nakashima et al., 2018; Ohmura et al., 2019; Hammarström et al., 2023; Nagai et al., 2024) and fewer patients who required dose reductions (OR = 0.49; 95% CI, 0.26–0.95; *I* ^2^ = 2%; *P =* 0.04, Figure 4B) (Kosaka et al., 2017; Nakashima et al., 2018; Ohmura et al., 2019; Gu et al., 2022; Nagai et al., 2024) compared to the control group. The low-dose regimen had almost significantly fewer patients who required a switch to a second-line regimen (OR = 0.55; 95% CI, 0.29–1.03; *I* ^2^ = 0%; *P* = 0.06, Figure 4C) (Kosaka et al., 2017; Nakashima et al., 2018; Ohmura et al., 2019; Hammarström et al., 2023).

## Discussion

In this systematic review and meta-analysis, we included seven studies and investigated the efficacy and safety of low-dose SMX-TMP treatment compared with a standard dose regimen. The results showed that low-dose SMX-TMP significantly reduced the mortality rate in patients with PJP (OR = 0.49, *P* = 0.004). Further subgroup and sensitivity analyses confirmed this finding, with the pooled ORs ranging from 0.0008 to 0.05. Low-dose SMX-TMP significantly reduced total AEs and improved the incidence of specific AEs (ORs ranged from 0.13 to 0.89), with statistically significant differences for rash, anemia, and hyponatremia (all *P* values < 0.001). Additionally, the low-dose group had significantly more patients who could complete the initial regimen and fewer patients who required dose reductions than the control group.

### Our results in comparison to previous reviews

Two previously published meta-analyses on using low-dose TMP-SMZ in patients with PJP examined the relationship between low-dose regimens and mortality and AEs (Tritle et al., 2021; Butler-Laporte et al., 2020). However, these two articles showed that a low-dose TMP-SMZ regimen did not improve patient mortality. This result may be because only three studies provided mortality outcomes in these two articles (Kosaka et al., 2017; Nakashima et al., 2018; Ohmura et al., 2019), which also prevented the authors from performing further sensitivity analyses, etc., to confirm the robustness of their results and to explore sources of heterogeneity. Similarly, the limited inclusion of studies prevented these two meta-analyses from exploring specific adverse events and, therefore, from fully assessing the impact of low-dose TMP-SMZ on the safety of patients with PJP (Tritle et al., 2021; Butler-Laporte et al., 2020).

In order to better understand the effect of low-dose TMP-SMZ on clinical outcomes in PJP patients, we conducted a comprehensive literature search. Our review included seven studies (Creemers-Schild et al., 2016; Kosaka et al., 2017; Nakashima et al., 2018; Ohmura et al., 2019; Gu et al., 2022; Hammarström et al., 2023; Nagai et al., 2024; Chang et al., 2016), which provided greater statistical power. The results suggest that a low-dose TMP-SMZ regimen was associated with significantly reduced mortality with a larger effect size (*P* = 0.004). We further explored potential influencing factors based on various study designs and clinical scenarios, and most of the sensitivity and subgroup analyses confirmed the robustness of our findings.

### Explain the results of our research

In this meta-analysis, the low-dose regimen significantly improved mortality and AEs. This finding may be due to several explanations. First, low-dose TMP-SMZ achieves adequate serum drug concentrations. Previous studies have demonstrated that low peak SMX concentrations are associated with treatment failure; 100–150 μg/mL serves as the optimal therapeutic range for PJP (Hughes et al., 1978), and peak SMX concentrations >200 μg/mL are associated with severe AEs (Klinker et al., 1998; Bowden et al., 1986). Although serum concentrations of SMX were not measured in the included studies, several previous studies have suggested that low-dose exposures are more consistent with the proposed target concentrations. Interestingly, in their study (N = 305), Dao et al. revealed that compared to the high-dose group (>15 mg/kg/d), the low-dose group (<15 mg/kg/d) had more patients (32% vs. 22%) in the optimal therapeutic concentration range attainment, while significantly fewer patients (29.3% vs. 75.7%) were above target (>150 μg/mL) (Dao et al., 2014). Thus, the low-dose regimen may provide adequate therapeutic effects while potentially avoiding the risk of more AEs.

Second, the current standard dosing regimen lacks evidence-based support. This regimen originated from a 1975 study of SMX-TMP in pediatric patients with cancer (Hughes et al., 1975). This study compared the clinical efficacy of 4–7 mg/kg/d and 20 mg/kg/d TMP-SMX in PJP and showed mortality rates of 33% (2/6) and 14.3% (2/14) in both groups (Hughes et al., 1975). Although there was no statistically significant difference, the 15–20 mg/kg/d TMP was subsequently used in adults and recommended by guidelines (Fishman et al., 2019; Mofenson et al., 2009). Thus, the standard dosing regimen ignores important differences in adult and pediatric pharmacokinetics and may be inappropriate for complex clinical scenarios in the modern era, such as advanced organ transplantation, immunosuppressive regimens, geriatric patients, and multiorgan failure.

Third, the low-dose regimen had better safety and tolerability. Our results showed that the low-dose regimen had a significantly lower incidence of total AEs than the standard-dose regimen (32.2% vs. 53.2.0%). Most of these dose-dependent AEs (e.g., rash, gastrointestinal disorders, myelosuppression, renal failure, hepatic disturbances, and electrolyte disturbances) are difficult to treat with supportive medications, which also makes it difficult to continue standard-dose regimen and affects patient prognosis. In their study, Medina found that 57% of patients with HIV PJP change from this treatment because of serious AEs (Medina et al., 1990). In contrast, the results of our meta-analysis suggested that the low-dose regimen had fewer patients requiring a switch to a second-line regimen (15.7% vs. 24.4%, Figure 4C) and more patients to complete the initial regimen (47.2% vs. 35.1%, Figure 4A) than the standard-dose regimen. In addition, fewer patients (15.7%) were switched to second-line drugs (e.g., atovaquone or pentamidine) in the low-dose regimen because of AEs than in the standard regimen (24.4%). However, findings from both HIV and non-HIV populations suggest that none of these alternative regimens are optimal or even as good as TMP-SMX (Smego et al., 2001; Koga et al., 2021). Therefore, we would speculate that the safety and good tolerability of the low dose of TMP-SMX may have contributed to the favorable results reported in our study.

### Current literature and future research

First, the definition of a low-dose regimen remains unclear. Some studies have suggested that 13.8 mg/kg/d of TMP may be the threshold for reducing severe AEs (Ohmura et al., 2019). Most studies included in this meta-analysis use 10 or 15 mg/kg/d as the low-dose threshold (Kosaka et al., 2017; Nakashima et al., 2018; Ohmura et al., 2019; Gu et al., 2022; Hammarström et al., 2023; Chang et al., 2016). Therefore, we performed a sensitivity analysis based on this and found that both threshold subgroups showed benefits in efficacy and safety. Future studies incorporating patient populations, renal function, and disease severity are needed to determine the optimal threshold for low-dose TMP-SMX therapy.

Second, only one small cohort study (N = 20) from the ICU setting was included in this meta-analysis, suggesting that PJP patients after renal transplantation who received the low-dose TMP-SMX regimen had comparable efficacy and fewer AEs than those who received the high-dose regimen (Gu et al., 2022). In order to further clarify whether critically ill patients could benefit from the low-dose regimen, we performed a subgroup analysis. We found that the low-dose regimen significantly reduced mortality (OR = 0.42, 95% CI 0.24–0.76) in PJP patients of the high-mortality prevalence subgroup (mortality rate>20%). However, because most included studies were non-ICU research, we could not obtain and evaluated the efficacy of the low-dose regimen based on adequate critical indicators, such as disease severity, oxygenation index, or respiratory support techniques, and further studies are needed to confirm our findings.

In addition, the two included studies reported that only 1.3% (1/77) (Kosaka et al., 2017) and 2.9% (4/136) (Nagai et al., 2024) of PJP patients received TMP-SMX prophylaxis. Therefore, PJP prophylaxis is essential in immunocompromised populations, especially in solid organ transplant recipients, rheumatic diseases, long-term hormone therapy, and bioimmunotherapy. TMP-SMX is the recommended first-line prophylaxis for PJP (Fishman et al., 2019; Mofenson et al., 2009). Depending on the patient’s condition, PJP prophylaxis often requires long-term or even lifelong use (Ghembaza et al., 2020; Kim et al., 2019).

### Limitations

Our meta-analysis has several limitations. The first is the potential selection bias due to the observational design of the included studies. An anticipated phase III randomized, placebo-controlled trial designed to compare the safety and efficacy of low-dose TMP-SMX with standard dose (10 vs. 15 mg/kg/day of TMP) for the treatment of PJP is currently underway (Sohani et al., 2022). Second, the small sample size of the studies may lead to more false-positive results. Also, some outcomes were evaluated by a small number of studies, so these results should be interpreted with caution. Third, there may have been confounding indications among the included cohorts (Nakashima et al., 2018; Nagai et al., 2024), but we obtained consistent results by both sensitivity and subgroup analyses. Fourth, we did not evaluate the effect of the administration of TMP-SMZ on the study results because there was insufficient data available. However, previous studies suggest that oral administration is almost completely absorbed and that there is similar drug distribution between routes of administration (Dao et al., 2014). Fifth, only one cohort focused on HIV-infected patients and only reported AEs (Chang et al., 2016). This may have limited the generalization of our study to HIV patients. Sixth, Finally, most of the included studies were from Asian populations (Kosaka et al., 2017; Nakashima et al., 2018; Ohmura et al., 2019; Gu et al., 2022; Nagai et al., 2024; Chang et al., 2016), which may limit the external validity of our results concerning various factors.

## Conclusion

In conclusion, our analysis has shown that low-dose TMP-SMX treatment significantly reduces mortality in patients with PJP. In addition, the low-dose regimen was associated with a significant reduction in adverse events, more patients completing initial treatment, and fewer patients requiring dose reductions or switching to a second-line regimen. It is important to consider the limitations, including the study design and the associated high risk of bias, which may contribute to a relatively low level of certainty in our findings. However, it is also important to acknowledge the promising nature of these results, as a low-dose TMP-SMX regimen has shown positive results in this patient population. Therefore, well-designed studies in this area are warranted.

## Data Availability

The original contributions presented in the study are included in the article/Supplementary Material, further inquiries can be directed to the corresponding authors.

## Abbreviations

CI, confidence interval; ICU, intensive care unit; LOS, length of stay; MD, mean difference; OR, odds ratio; RCTs, randomized controlled trials; SD, standard deviations.
