# Supplementary material for: Low-dose trimethoprim-sulfamethoxazole treatment for Pneumocystis pneumonia: a systematic review and meta-analysis
Source: Front Pharmacol. 2024 Nov 13;15:1422490. doi: 10.3389/fphar.2024.1422490 (PMC11598433; doi:10.3389/fphar.2024.1422490)
Supplement: Supplementary file 1 [file DataSheet1.docx]

**Low-Dose Trimethoprim-Sulfamethoxazole Treatment for Pneumocystis Pneumonia: a systematic review and meta-analysis**

*Corresponding author: Hui-Bin Huang,

Email: [psyc6789@163.com](mailto:psyc6789@163.com).

**Additional files**

Additional file 1 PRISMA checklist……………………………………………………………………………………………………………………………………………………………………………………………….……………………………………..2

Additional file 2 Search Strategy………………………………………………………………………………………………………………………………………………………………………………………………………………….……………………..5

Additional file 3 Studies needed for review but not included in the current meta-analysis ………………………………………………………………………………..…………………………..………….……………….……..7

Additional file 4 Assessment of observational studies……………………….……………………………………………….…………………………………………………………………………………………………………………....…….….9

Additional file 5 Funnel plot of comparison: short-term mortality…..…………………………………………………………………….…………………………………………………………….…………………………………....………10

Additional file 6 Summary of adverse events of the low-dose group in the included studies……………………………………………………………...…………………………………………………………………....……….11

**Additional File 1 PRISMA 2009 checklist**

| **Section/topic** | **#** | **Checklist item** | **Reported on page #** |
| --- | --- | --- | --- |
| **TITLE** | | |  |
| Title | 1 | Identify the report as a systematic review, meta-analysis, or both. |  |
| **ABSTRACT** | | |  |
| Structured summary | 2 | Provide a structured summary including, as applicable: background; objectives; data sources; study eligibility criteria, participants, and interventions; study appraisal and synthesis methods; results; limitations; conclusions and implications of key findings; systematic review registration number. |  |
| **INTRODUCTION** | | |  |
| Rationale | 3 | Describe the rationale for the review in the context of what is already known. |  |
| Objectives | 4 | Provide an explicit statement of questions being addressed with reference to participants, interventions, comparisons, outcomes, and study design (PICOS). |  |
| **METHODS** | | |  |
| Protocol and registration | 5 | Indicate if a review protocol exists, if and where it can be accessed (e.g., Web address), and, if available, provide registration information including registration number. |  |
| Eligibility criteria | 6 | Specify study characteristics (e.g., PICOS, length of follow-up) and report characteristics (e.g., years considered, language, publication status) used as criteria for eligibility, giving rationale. |  |
| Information sources | 7 | Describe all information sources (e.g., databases with dates of coverage, contact with study authors to identify additional studies) in the search and date last searched. |  |
| Search | 8 | Present full electronic search strategy for at least one database, including any limits used, such that it could be repeated. |  |
| Study selection | 9 | State the process for selecting studies (i.e., screening, eligibility, included in systematic review, and, if applicable, included in the meta-analysis). |  |
| Data collection process | 10 | Describe method of data extraction from reports (e.g., piloted forms, independently, in duplicate) and any processes for obtaining and confirming data from investigators. |  |
| Data items | 11 | List and define all variables for which data were sought (e.g., PICOS, funding sources) and any assumptions and simplifications made. |  |
| Risk of bias in individual studies | 12 | Describe methods used for assessing risk of bias of individual studies (including specification of whether this was done at the study or outcome level), and how this information is to be used in any data synthesis. |  |
| Summary measures | 13 | State the principal summary measures (e.g., risk ratio, difference in means). |  |
| Synthesis of results | 14 | Describe the methods of handling data and combining results of studies, if done, including measures of consistency (e.g., I^2^) for each meta-analysis. |  |

| Risk of bias across studies | 15 | Specify any assessment of risk of bias that may affect the cumulative evidence (e.g., publication bias, selective reporting within studies). |  |
| --- | --- | --- | --- |
| Additional analyses | 16 | Describe methods of additional analyses (e.g., sensitivity or subgroup analyses, meta-regression), if done, indicating which were pre-specified. |  |
| **RESULTS** | | |  |
| Study selection | 17 | Give numbers of studies screened, assessed for eligibility, and included in the review, with reasons for exclusions at each stage, ideally with a flow diagram. |  |
| Study characteristics | 18 | For each study, present characteristics for which data were extracted (e.g., study size, PICOS, follow-up period) and provide the citations. |  |
| Risk of bias within studies | 19 | Present data on risk of bias of each study and, if available, any outcome level assessment (see item 12). |  |
| Results of individual studies | 20 | For all outcomes considered (benefits or harms), present, for each study: (a) simple summary data for each intervention group (b) effect estimates and confidence intervals, ideally with a forest plot. |  |
| Synthesis of results | 21 | Present results of each meta-analysis done, including confidence intervals and measures of consistency. |  |
| Risk of bias across studies | 22 | Present results of any assessment of risk of bias across studies (see Item 15). |  |
| Additional analysis | 23 | Give results of additional analyses, if done (e.g., sensitivity or subgroup analyses, meta-regression [see Item 16]). |  |
| **DISCUSSION** | | |  |
| Summary of evidence | 24 | Summarize the main findings including the strength of evidence for each main outcome; consider their relevance to key groups (e.g., healthcare providers, users, and policy makers). |  |
| Limitations | 25 | Discuss limitations at study and outcome level (e.g., risk of bias), and at review-level (e.g., incomplete retrieval of identified research, reporting bias). |  |
| Conclusions | 26 | Provide a general interpretation of the results in the context of other evidence, and implications for future research. |  |
| **FUNDING** | | |  |
| Funding | 27 | Describe sources of funding for the systematic review and other support (e.g., supply of data); role of funders for the systematic review. |  |

**Appendix 2 Search Strategy**

Database: 3 databases

Search completed March 10, 2024

----------------------------------------------------------------------------------------------------------------------

**PubMed**

(((((trimethoprim/sulfamethoxazole[Title/Abstract]) OR (TMP-SMX[Title/Abstract])) OR (TMP/SMX[Title/Abstract])) OR ("Trimethoprim, Sulfamethoxazole Drug Combination"[Mesh])) OR (co-trimoxazole[Title/Abstract])) AND (((((((Pneumocystis jirovecii pneumonia[Title/Abstract]) OR (Pneumocystis carinii pneumonia[Title/Abstract])) OR (Pneumocystis[Title/Abstract])) OR (PJP, pneumonia[Title/Abstract])) OR (PCP, pneumonia[Title/Abstract])) OR ("Pneumonia, Pneumocystis"[Mesh])) AND (((dose[Title/Abstract]) OR (dosing[Title/Abstract])) OR (dosage[Title/Abstract]))))

**Embase**

No. Query

#16 #4 AND #10 AND #15

#15 #12 OR #13 OR #14

#14 'dosage':ab,ti AND ([embase]/lim OR [medline]/lim)

#13 'dosing':ab,ti AND ([embase]/lim OR [medline]/lim)

#12 'dose':ab,ti AND ([embase]/lim OR [medline]/lim)

#11 #5 OR #6 OR #7 OR #8 OR #9 OR #10

#10 'cotrimoxazole'/exp

#9 'co-trimoxazole':ab,ti AND ([embase]/lim OR [medline]/lim)

#8 'smx-tmp':ab,ti AND ([embase]/lim OR [medline]/lim)

#7 'tmp/smx':ab,ti AND ([embase]/lim OR [medline]/lim)

#6 'tmp-smx':ab,ti AND ([embase]/lim OR [medline]/lim)

#5 'trimethoprim/sulfamethoxazole':ab,ti AND ([embase]/lim OR [medline]/lim)

#4 #1 OR #2 OR #3

#3 'pneumocystis pneumonia'/exp

#2 'pneumocystis':ab,ti AND ([embase]/lim OR [medline]/lim)

#1 'pneumocystis jirovecii pneumonia':ab,ti AND ([embase]/lim OR [medline]/lim)

**Cochrane library**

ID Search#1

#1 ("pneumocystis jirovecii pneumonia "):ti,ab,kw (Word variations have been searched)

#2 ("pneumocystis"):ti,ab,kw (Word variations have been searched)

#3 ("Pneumocystis carinii pneumonia "):ti,ab,kw (Word variations have been searched)

#4 ("pneumocystis pneumonia "):ti,ab,kw (Word variations have been searched)

#5 #1 OR #2 OR #3 OR #4

#6 ("trimethoprim/sulfamethoxazole"):ti,ab,kw (Word variations have been searched)

#7 ("co-trimoxazole"):ti,ab,kw (Word variations have been searched)

#8 ("tmp-smx"):ti,ab,kw (Word variations have been searched)

#9 ("smx-tmp"):ti,ab,kw (Word variations have been searched)

#10 #6 OR #7 OR #8 OR #9

#11 ("prophylactic"):ti,ab,kw (Word variations have been searched)

#12 ("prophylaxis"):ti,ab,kw (Word variations have been searched)

#13 ("prevent"):ti,ab,kw (Word variations have been searched)

#14 ("prevention"):ti,ab,kw (Word variations have been searched)

#15 #11 OR #12 OR #13 OR #14

#16 #5 AND #10 AND #15

**Additional File 3**

**Table. Studies needed for full-reviewed but not included in the current meta-analysis (n=12 articles)**

| No | Study |
| --- | --- |
| 1 | Creemers-Schild D, Kroon FP, Kuijper EJ, de Boer MG. Treatment of Pneumocystis pneumonia with intermediate-dose and step-down to low-dose trimethoprim-sulfamethoxazole: lessons from an observational cohort study. Infection. 2016 Jun;44(3):291-9. |
| 2 | Ji J, Wang Q, Huang T, Wang Z, He P, Guo C, Xu W, Cao Y, Dong Z, Wang H. Efficacy of Low-Dose Trimethoprim/Sulfamethoxazole for the Treatment of Pneumocystis jirovecii Pneumonia in Deceased Donor Kidney Recipients. Infect Drug Resist. 2021 Nov 24;14:4913-4920. |
| 3 | Thomas M, Rupali P, Woodhouse A, Ellis-Pegler R. Good outcome with trimethoprim 10 mg/kg/day-sulfamethoxazole 50 mg/kg/day for Pneumocystis jirovecii pneumonia in HIV infected patients. Scand J Infect Dis. 2009;41(11-12):862-8. |
| 4 | Sonomoto K, Tanaka H, Nguyen TM, Yoshinari H, Nakano K, Nakayamada S, Tanaka Y. Prophylaxis against pneumocystis pneumonia in rheumatoid arthritis patients treated with b/tsDMARDs: insights from 3787 cases in the FIRST registry. Rheumatology (Oxford). 2022 May 5;61(5):1831-1840. |
| 5 | Sohani ZN, Butler-Laporte G, Aw A, Belga S, Benedetti A, Carignan A, Cheng MP, Coburn B, Costiniuk CT, Ezer N, Gregson D, Johnson A, Khwaja K, Lawandi A, Leung V, Lother S, MacFadden D, McGuinty M, Parkes L, Qureshi S, Roy V, Rush B, Schwartz I, So M, Somayaji R, Tan D, Trinh E, Lee TC, McDonald EG. Low-dose trimethoprim-sulfamethoxazole for the treatment of Pneumocystis jirovecii pneumonia (LOW-TMP): protocol for a phase III randomised, placebo-controlled, dose-comparison trial. BMJ Open. 2022 Jul 21;12(7):e053039. |
| 6 | Ice LL, Barreto JN, Dao BD, Wolf RC, Dierkhising RA, Jannetto PJ, Langman LJ, Tosh PK. Relationship of Sulfamethoxazole Therapeutic Drug Monitoring to Clinical Efficacy and Toxicity: A Retrospective Cohort Study. Ther Drug Monit. 2016 Jun;38(3):319-26. |
| 7 | Shibata T, Tonooka K, Tsuchida K, Mitomi H, Shibata T, Katsuyama N. [Retrospective investigation of side effects and prognoses of moderate-dose trimethoprim-sulfamethoxazole treatment for pneumocystis pneumonia that developed in patients with autoimmune diseases]. Nihon Rinsho Meneki Gakkai Kaishi. 2016;39(3):213-8. Japanese. |
| 8 | Yamanaga S, Tanaka K, Kinoshita K, Kaba A, Fujii M, Ogata M, Hidaka Y, Kawabata C, Toyoda M, Uekihara S, Kashima M, Miyata A, Inadome A, Yokomizo H. Impact of Very Low-Dose Trimethoprim-Sulfamethoxazole on Serum Creatinine after Renal Transplantation: A Retrospective Study. Transplant Proc. 2020 Jul-Aug;52(6):1757-1761. |
| 9 | Haseeb A, Abourehab MAS, Almalki WA, Almontashri AM, Bajawi SA, Aljoaid AM, Alsahabi BM, Algethamy M, AlQarni A, Iqbal MS, Mutlaq A, Alghamdi S, Elrggal ME, Saleem Z, Radwan RM, Mahrous AJ, Faidah HS. Trimethoprim-Sulfamethoxazole (Bactrim) Dose Optimization in Pneumocystis jirovecii Pneumonia (PCP) Management: A Systematic Review. |
| 10 | Alshehri S, Alghuraybi R, Ayoub E, Bokhary J, Lashkar M, Alshibani M, Eljaaly K. Evaluation of Weight-Based Co-trimoxazole Dosing in a Saudi Tertiary Hospital. Cureus. 2023 Oct 20;15(10):e47400. doi: 10.7759/cureus.47400. PMID: 38022178; PMCID: PMC10657735. |

**Additional file 4**

**Table Definitions and outcomes of mortality and definitions of low-dose TMP of each study among the included studies.**

| Study | Study sample size | | 30-day mortality | | 90-day mortality | | 180-day mortality | | Definition of low-dose SMZ-TMP | Standard dose of SMZ-TMP |
| --- | --- | --- | --- | --- | --- | --- | --- | --- | --- | --- |
|  | LD | SD | LD | SD | LD | SD | LD | SD | LD | SD |
| Chang 2016 [26] | 25 | 27 |  |  |  |  |  |  | <15 mg/kg/d of TMP | >15 mg/kg/d |
| Gu 2022 [15] | 10 | 10/10 | 0 | 2 |  |  |  |  | 8 mg/kg/d TMP | 15 mg/kg/d |
| Hammarström 2023 | 80 | 33 | 11 | 5 |  |  |  |  | 7.5-15 mg/kg/d | 15-20 mg/kg/d |
| Kosaka 2017 [29] | 41 | 36 |  |  | 8 | 9 |  |  | <15 mg/kg/d | 15-20 mg/kg/d |
| Nagai 2024 [14] | 55 | 81 | 4 | 14 |  |  | 10 | 23 | <12.5 mg/kg/d | 12.5-20 mg/kg/d |
| Nakashima 2018 [32] | 24 | 29 | 1 | 9 |  |  | 2 | 14 | 4-10 mg/kg/d | 10-20 mg/kg/d |
| Ohmura 2019 | 52 | 29 | 2 | 1 |  |  | 3 | 3 | ≤10-15 mg/kg/d | 15-20 mg/kg/d |

LD = low dose regimen; SD = standard dose regimen.

**Additional file 5**

**Table.** **Summarized the severity of respiratory failure and the associated respiratory supports in the included studies.**

| Study | N | | PaO_2_/FiO_2_ | | Without O_2_, % | | O_2_ therapy, n(%) | | HFNC, n(%) | | NIV, n(%) | | IMV, n(%) | |
| --- | --- | --- | --- | --- | --- | --- | --- | --- | --- | --- | --- | --- | --- | --- |
|  | **LD** | **SD** | **LD** | **SD** | **LD** | **SD** | **LD** | **SD** | **LD** | **SD** | **LD** | **SD** | **LD** | **SD** |
| Chang 2016 | 25 | 27 |  |  |  |  |  |  |  |  |  |  |  |  |
| Gu 2022 | 10 | 10 | 148 | 146.5 |  |  |  |  | 8(80) | 9(90) |  |  | 0(0) | 0(0) |
| Hammarstrom 2023 | 80 | 33 | 216 | 195 |  |  |  |  |  |  | 12(15) vs 9(27)# | | | |
| Kosaka 2017 | 41 | 36 | 223.9 | 238.6 |  |  |  |  |  |  |  |  | 14 (34.1) | 11 (30.6) |
| Nagai 2024 | 55 | 81 |  |  |  |  |  |  | 0(0) | 1(1.2) | 0(0) | 1(1.2) | 0(0) | 0(0) |
| Nakashima 2018 | 24 | 29 |  |  | 9(37.5) | 7(24.1) | 13(54.2) | 14(48.3) | 2(8.3) | 0(0) |  |  |  |  |
| Ohmura 2019 | 52 | 29 |  |  | 9(30) vs 13(44.8)& | | | |  |  |  |  |  |  |

# A total of 14 and 9 patients received NIV or IMV in the LD and SD groups, respectively.

& A total of 9 and 13 patients received treatment of Oxygen >5 L/min in the LD and SD groups, respectively.

**Table S6: Quality assessment and overall risk of bias of included studies**

| First author / year | Patient selection | | | | Comparability | Outcome | | | Risk of bias |
| --- | --- | --- | --- | --- | --- | --- | --- | --- | --- |
|  | Representation of the exposed cohort | Selection of the non-exposed cohort | Ascertainment of exposure | Outcome of  interest not  present at start | Comparability of cohorts on the basis of the design or analysis | Assessment  of outcome | Was follow-up long enough for outcomes to occur | Adequacy of follow up of cohorts |  |
| Chang 2016 | ★ | ★ | ★ | ☆ | ★★ | ★ | ★ | ★ | 8 |
| Gu 2022 | ★ | ★ | ★ | ☆ | ★★ | ★ | ★ | ★ | 8 |
| Hammarström 2023 | ★ | ★ | ★ | ☆ | ★★ | ★ | ★ | ★ | 8 |
| Kosaka 2017 | ★ | ★ | ★ | ☆ | ★★ | ★ | ★ | ★ | 8 |
| Nagai 2024 | ★ | ★ | ★ | ☆ | ★★ | ★ | ★ | ★ | 8 |
| Nakashima 2018 | ★ | ★ | ★ | ☆ | ★★ | ★ | ★ | ★ | 8 |
| Ohmura 2019 | ★ | ★ | ★ | ☆ | ★★ | ★ | ★ | ★ | 8 |

H=high quality; M=moderate quality; L= low quality.

**Additional File 6**

**Funnel plot of comparison: Discontinuation rate**


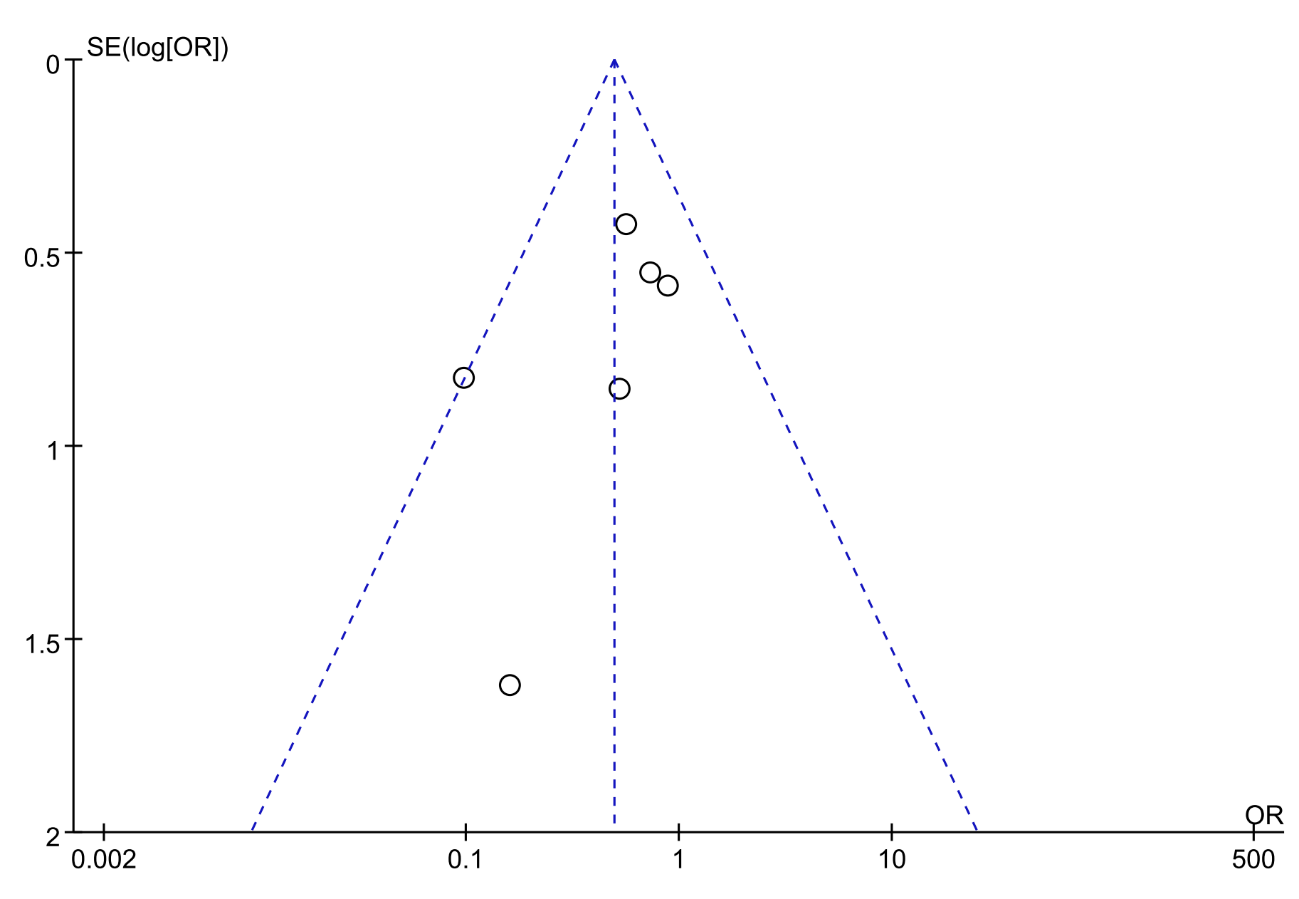


**Additional file 7**

**Table. Summary of adverse events of the low-dose group and standard-dose group in the included studies**

| Study |  | A | | B | | C | | D | | E | | F | | G | | H | | I | | J | | K | |
| --- | --- | --- | --- | --- | --- | --- | --- | --- | --- | --- | --- | --- | --- | --- | --- | --- | --- | --- | --- | --- | --- | --- | --- |
|  | LD/SD | LD | SD | LD | SD | LD | SD | LD | SD | LD | SD | LD | SD | LD | SD | LD | SD | LD | SD | LD | SD | LD | SD |
| Chang 2016 [26] | 25/27 |  |  |  |  |  |  |  |  | 7 | 15 | 21 | 35 | 7 | 5 | 4 | 6 |  |  |  |  | 7 | 14 |
| Gu 2022 [15] | 10/10 | 0 | 0 |  |  | 2 | 7 | 1 | 6 | 3 | 9 | 2 | 5 |  |  | 0 | 0 | 0 | 0 | 0 | 0 |  |  |
| Hammarström 2023 | 80/33 |  |  |  |  |  |  |  |  |  |  |  |  |  |  |  |  |  |  |  |  | 20 | 11 |
| Kosaka 2017 [29] | 41/36 | 1 | 3 |  |  | 2 | 1 | 0 | 1 | 1 | 6 | 0 | 1 | 1 | 4 | 5 | 4 | 0 | 2 | 0 | 0 | 7 | 15 |
| Nagai 2024 [14] | 55/81 | 16 | 46 | 0 | 8 |  |  | 0 | 2 | 2 | 2 | 2 | 3 | 4 | 23 | 3 | 12 |  |  |  |  | 16 | 46 |
| Nakashima 2018 [32] | 24/29 | 3 | 3 |  |  |  |  |  |  | 2 | 3 | 6 | 6 | 4 | 10 | 3 | 7 |  |  | 3 | 2 | 14 | 21 |
| Ohmura 2019 | 22/30/29 | 11 | 7 | 5 | 4 | 0 | 1 | 0 | 3 |  |  | 4 | 1 | 11 | 8 | 3 | 3 |  |  | 12 | 11 | 25 | 18 |

A = skin rashes; B = Nausea; C = leukopenia; D = anemia; E = thrombocytopenia; F = increased ALT levels; G = hyponatremia; H = hyperkalemia; I = anorexia; J = renal injury; K=total adverse events.

LD = low dose regimen; SD = standard dose regimen.
